# Analytical microscopy techniques using coaxial and oblique illuminations to detect thin glass particulates generated from glass vials for parenteral drug products

**DOI:** 10.1186/s42649-024-00101-3

**Published:** 2024-10-23

**Authors:** Adedayo M. Sanni, Adedamola A. Opalade, Armen Shamirian, Spencer Mattson, Eric Driscoll, Michael St. Martin, Shikhar Mohan, Brooke Trimmer, Tarq Bunch, Robert Ovadia, Jungjoo Yoon, Sarina Ma, Chris Foti

**Affiliations:** 1 Analytical Development and Operations, Gilead Sciences Inc, Foster City, CA 94404 USA; 2 ORIC Pharmaceuticals Inc., South San Francisco, CA 94080 USA; 3 Development and Technical Services, Gilead Sciences Inc, La Verne, CA 91750 USA; 4 Process Technologies and Development, Gilead Sciences Inc, Foster City, CA 94404 USA; 5 Pharmaceutical Development and Technology, Gilead Sciences Inc, Foster City, CA 94404 USA

**Keywords:** Glass delamination, Parenteral drugs, Optical microscopy, Microspectroscopy, Lamellae, Coaxial illumination, Oblique illumination, Thin-film interference

## Abstract

Glass vials are the most widely used primary containers for the packaging of parenteral products due to their optical clarity, general inertness, and hermetic properties, but under certain circumstances, they can pose safety concerns. Most of these issues are related to the potential formation of glass particulates through delamination or precipitation, resulting from the chemical interaction between the drug product and the inner surface of the glass vial. Hence, it is imperative for pharmaceutical companies to conduct product-vial compatibility studies to determine the appropriate packaging/container closure system. To support this development activity, scientists need to develop analytical methods to detect subvisible glass particulates in parenteral products, along with the appropriate positive controls, to facilitate detection and identification. This paper outlines the utilization of coaxial/episcopic and oblique illumination microscopy, combined with spectroscopic techniques, to detect thin glass particulates generated from a modified procedure. It also showcases the importance of angle-dependent lighting in visualizing positive control samples containing thin glass particulates. The analytical microscopy techniques discussed in this paper can assist scientists in selecting suitable container closure systems for developing parenteral products.

**Supplementary Information:**

The online version contains supplementary material available at 10.1186/s42649-024-00101-3.

## Introduction

Glass vials have been used extensively as a primary packaging container for parenteral products (Sacha et al. 2010). They are desirable for pharmaceutical applications due to their hermeticity, chemical durability, and relative ease of sterilization and depyrogenation. Properties such as transparency, recyclability, and ability to withstand thermal exposure are other factors that contribute to their preferential usage as packaging containers (Sacha et al. 2010; Schaut et al. 2017).

Although the benefits of glass vials are evident, a well-known limitation is the potential interaction between the formulated product and the glass container. Such an interaction can lead to the generation of thin glass particulates through (1) delamination-associated corrosion or (2) precipitation from elements in solution (Ma et al. 2021). These limitations compromise product quality by generating thin glass particulates and are typically referred to as “failure modes of the vial” (Ma et al. 2021; Srinivasan et al. 2019). Glass particulates in parenteral drug products has been subject to scrutiny by the FDA (FDA 2011), discussed widely in the USP (USP 2013), and is an increasingly popular topic in the literature (Sacha et al. 2010; Srinivasan et al. 2019; Zarour-Shalev et al. 2015). Thus, it is crucial to develop analytical methods capable of identifying glass particulates to support container selection and develop the right control strategy to ensure the drug product’s safety.

Factors impacting the vial failure modes include the chemical composition (USP 2013; Ditter et al. 2018; Rayaprolu et al. 2017) of the glass vial, manufacturing conditions (USP 2013; Ditter et al. 2018; Ditter et al. 2018; USP. 2023)), nature of the drug product formulation, and procedures for vial handling (Sloey et al. 2013; Iacocca et al. 2010). Various methods in the literature have been employed to detect thin (typically < 1 μm thick) glass particulates generated from parenteral glass vials. The most common approaches include: a combination of mechanical swirling and visual inspection for reflective particulates (twinkling) (Sloey et al. 2013), flow imaging (Ditter et al. 2018)and electron microscopy (Ma et al. 2021; Schumacher et al. 2015). However, these methods are prone to drawbacks unique to each technique. To date, the most successful technique adopted in identifying thin, subvisible glass particulates is electron microscopy. However, challenges such as the complex instrument operation, availability of trained scientists, and time-consuming sample preparation persist. Consequently, there is a need for a selective, and robust analytical technique that is complimentary to electron microscopy, useful in laboratories to identify glass particulates that have submicron (< 1 μm) thickness which is comparable in size to the wavelength of visible light.

Optical microscopy (OM) is an accessible and selective technique found in analytical laboratories that can differentiate glass particulates from other non-glass particulates. In OM, using the appropriate lighting (transmitted, coaxial, or oblique) for specific applications allow scientists to distinguish between various particles (McCrone 2024). The thin nature of glass particulates from glass delamination or precipitation allows the scientists to uniquely detect them by taking advantage of their interaction with light under the optical microscope. Such interaction leads to the generation of colored gradient, often called thin-film interference (McCrone 2024; Delly 2019). This interference phenomenon is same as those commonly observed in soap bubbles (P.P. 2012). The materials exhibiting thin-film interference show thickness dependent color gradient, as described in the literature (Urone 2012; –Zhang et al. 2021). Multiple OM illumination configurations (e.g. coaxial and oblique illumination) can be leveraged for detecting and differentiating various types of particulates as recommended in compendial documents (USP. 2024; USP. 2013). Despite these recommendations, there is limited work on using thin-film interference in OM in the detection of thin (< 1 μm thick) glass particulates from different vial sources.

In this paper, we demonstrate that OM coupled with scanning electron microscopy -energy dispersive X-ray spectroscopy (SEM-EDS), Laser-induced Breakdown Spectroscopy (LIBS), and Fourier transform infrared (FTIR) spectroscopy are useful analytical methods to evaluate and monitor glass container compatibility with the drug product. The OM configuration setup described was used to detect thin glass particulates generated from stressed vials with two different glass compositions undergoing differing failure modes. The microscopy detection was achieved through the combination of both coaxial and oblique illumination. Our study confirmed that thin glass particulates can be detected under coaxial illumination due to thin-film interference resulting in a colored image but are not visible under oblique illumination. We note that within our instrument configuration, thin-film interference is fully observable under coaxial illumination and becomes non-observable when the angle of incidence falls between 15–60º (i.e., oblique). This appearance and disappearance of thin-film interference criteria is useful during evaluation of compatibility with drug products.

## Materials and methods

### Materials

Sodium hydroxide (Fisher Scientific, NJ, USA), glycine (J.T Baker, PA, USA), glutaric acid (Aldrich, MO, USA) were used to prepare various pH solutions in this study.

The vials used in this study are 12 mL vials, Vial A, sourced from vendor A and 2R ready to use (RTU) vials, Vial B, sourced from vendor B. Accessories such as 13 mm aluminum vial caps (West Pharmaceutical Services Inc, PA, USA ), rubber stoppers (West Pharmaceutical Services Inc, PA, USA ), 0.6 μm Cytiva polycarbonate “Nucleopore” membrane filters (Global Life Sciences Solutions, Buckinghamshire, UK), Acrodisc 0.2 μm PVDF syringe filter, Analyslide Petri dishes (Pall Corporation, MI, USA) were used in this study. All materials were used as received.

### Experimental design

Two types of glass vials were selected and sourced from different vendors based on their distinct failure modes as assessed by SEM-EDS and in line with the previously reported work by Ma et al. ((Ma et al. 2021). Irrespective of the type of failure mode, OM was utilized to detect the thin glass particulates produced from both vial categories. This detection was achieved using two distinct microscope lighting configurations: Coaxial and oblique. Using particulates generated from one of the vial types, angle of incidence-dependent OM, LIBS and FTIR microspectroscopic studies were performed to provide additional insight on the applicability and the robustness of the reported detection method to the detection of glass particulates from different sources.

### Experimental methods

#### Particulate generation from glass vials

##### Vial A

To generate glass particulates from Vial A, an earlier method developed by Sloey and coworkers (Sloey et al. 2013) was slightly modified to induce delamination. To mimic pharmaceutical washing and depyrogenation, 200 µL of purified water was added to Vial A, then the vial was heated on a hot plate (IKA, 5030001, RET control-visc S001, NC, USA) at 250 °C for ca. 15 to 90 min followed by cooling to room temperature for at least 30 min. Approximately 1 mL of 20 mM glycine (pH 10.0) solution filtered through a 0.2 μm syringe filter was added to each cooled vial. The filled vials were capped and stored in an oven chamber (Fisherbrand Isotemp, Thermo Scientific, Lagenselbold, Germany) set to 50 °C for ca. 24 h. After storage, the vial contents were visually inspected for twinkling particulates. The vial contents were then filtered through a 0.6 μm polycarbonate filter, air-dried in a particle-free environment and stored in a Petri dish to air-dry before analytical assessment.

##### Vial B

The method described by Ma and coworkers (Ma et al. 2021) was followed to generate particulates in Vial B. Specifically, 2.1 mL of 76 mM sodium glutarate (pH 11.6) solution, filtered through a 0.2 μm syringe filter was transferred into Vial B. Next, the vial was closed with a rubber stopper, crimped with an aluminum cap, and placed in an oven chamber set to 60 °C. Twinkling particulates were obtained within three weeks and allowed to stay in the stability chamber until needed for filtration. The vial contents were filtered through a 0.6 μm polycarbonate membrane filter (a gold polycarbonate membrane fileter was used to prepare samples for FTIR analysis), air-dried in a particle-free environment, and stored in a Petri dish to air-dry before analytical assessment.

#### Instrumentation

##### Scanning electron microscopy- energy dispersive spectroscopy (SEM-EDS)

The morphology of the particulates obtained by filtration and the texture of the inner wall of the glass vials were evaluated by SEM-EDS. A portion of the polycarbonate filter membranes containing filtered particulates were cut out and transferred to an SEM specimen stub using a double-sided carbon tape. This mounted specimen was sputter coated using a Cressington 108 Auto coater (Cressignton Scientific Instruments, Watford, United Kingdom) with Au-Pd target, and then analyzed using a JEOL JSM-IT500HR (Jeol Ltd, Tokyo, Japan) field emission SEM (FE-SEM). To assess the interior surface of the stressed vials, thoroughly rinsed vials were sectioned using a TechCut 4x Allied High Tech cutter (Allied High Tech products, Inc, California, United States of America ), followed by cleaning of the sectioned vial portions with water, tween 80 solution, 70% isopropanol polyester wipe, and then air dried. The cleaned sectioned vials were mounted onto an SEM specimen stub using double-sided carbon tape and sputter coated using a Cressington 108 coater with Au-Pd target, and then analyzed using a JEOL JSM-IT500HR FE-SEM after performing daily check analysis using Cu standard. Peaks due to Au/Pd were removed from the EDS spectrum as they were introduced during sample preparation step from sputter coating.

##### Optical microscopy (OM)

**Fig. 1:**
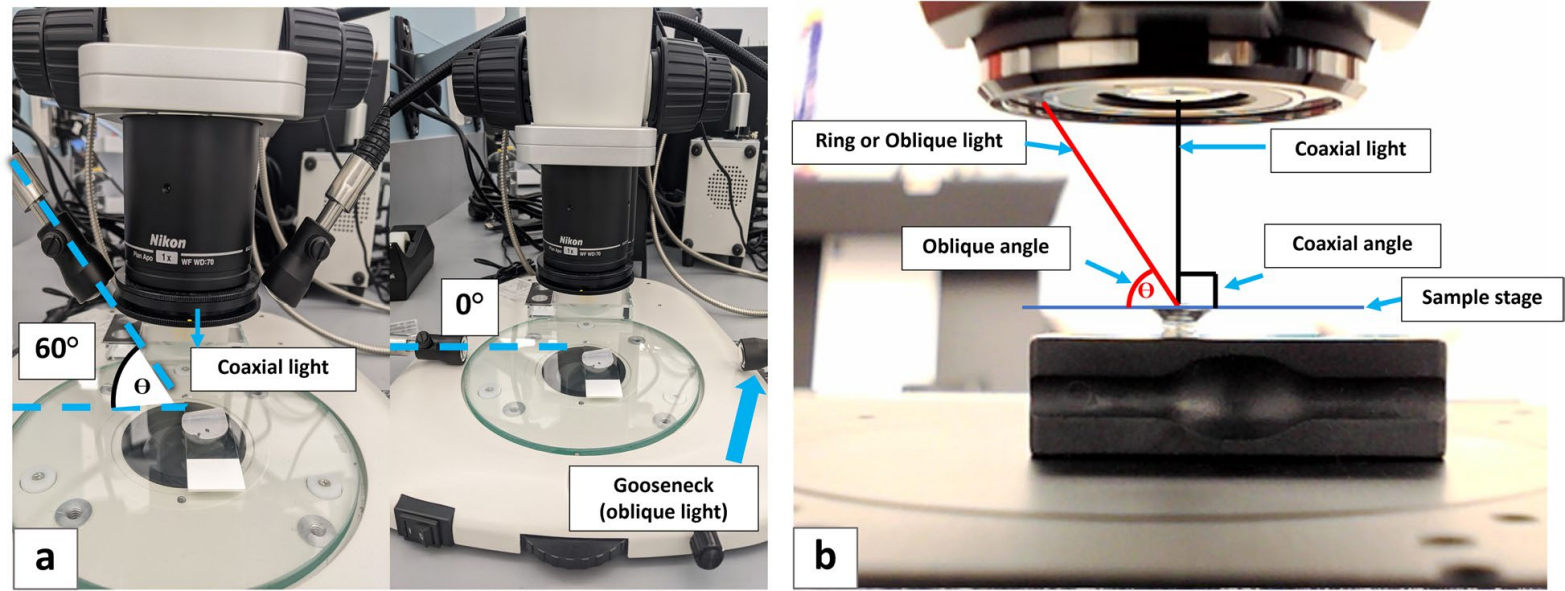
**a** Experimental setup on the stereomicroscope showing both oblique (gooseneck) and coaxial illumination in addition to the AOI (θ). **b** Experimental setup on the Keyence microscope showing both oblique (ring) and coaxial illumination with the AOI (θ) indicated

OM studies were performed on the polycarbonate filter membrane following the filtration of the vial contents, as described in the Experimental Methods Section. The studies were performed using a Nikon SMZ1270 stereomicroscope (Nikon Corp, Tokyo, Japan) and a Keyence VHX-7000 digital microscope (Keyence corporation, Osaka, Japan); both microscopes were equipped with coaxial and oblique (or “ring”) illumination. The membrane filters were examined for the presence of thin particulates, exhibiting thin-film interference, possessing sharp and well-defined edges using coaxial and ring (oblique) illumination under the microscope. Studies on the incident angle dependency of the light source to detect thin-film interference colors was carried out using Nikon SMZ1270 stereomicroscope equipped with coaxial and gooseneck (oblique) lighting and a goniometer. To determine the illumination angle necessary for the detection and visualization of glass particulates, a goose neck lighting (oblique illumination) source was adjusted between 15–60° during the observation of the filter under the stereomicroscope using the experimental setup shown in Fig. 1a. Angle dependent microscopy analysis was also conducted on the Keyence digital microscope using the coaxial illumination and the ring lighting as the oblique illumination source (Fig. 1b). Each objective on the digital microscope allowed different angles of incidence.

##### Laser-induced breakdown spectroscopy (LIBS)

LIBS analysis was carried out using a Keyence VHX7000 digital microscope equipped with an elemental analyzer and a 355 nm laser with 10 μm spot size. The filter containing the glass particulates was placed on the microscope stage and brought into focus with the aid of the coaxial light using the thin-film interference exhibited by thin glass particulates as a visual guide. The atomic/weight% of elements present in the particulates was recorded.

##### Fourier transform infrared (FTIR) micro-spectroscopy

A Continuum™ infrared microscope with a mercury cadmium telluride (MCT/A) detector interfaced with a Magna-IR 560^®^ FTIR spectrometer (Thermo Scientific Instruments, Wisconsin, United States of America ) was used to analyze the thin particulates from Vial B. The microscope was operated in the transmission mode for the analysis. The background spectrum was collected through a neat KBr spot. 128 sample scans were collected from 650 to 4000 cm^−1^ at a spectral resolution of 4 cm^−1^. Additionally, mosaic images of the filter containing glass particulates were acquired using Nicolet FTIR RaptIR microscope (Thermo Fisher Scientific, Wisconsin, United States of America), equipped with both the 4x and 15x microscope objectives. The spectral acquisition on the RaptIR microscope was achieved using both the reflection mode and the micro-attenuated total reflectance (micro-ATR) module with a germanium tip. In the case of the ATR mode, the background spectrum was first acquired on the portion of the membrane filter that contained no glass particulate. The ATR tip was then placed on a portion of the filter containing the glass particulate on the membrane filter (sample). The spectrum of the glass particulate was ultimately obtained by subtracting the background from the sample spectrum.

## Results and discussion

### Glass particulate generation

Glass particulates were generated in both types of vials as detailed in the Experimental Methods. For Vial A, a slight modification of the method developed by Sloey and coworkers (Sloey et al. 2013) was implemented to generate glass particulates. Since the heel region of the vials is typically the most susceptible portion of glass vials for failure, subjecting this region to more intense heat may result in an extensive damage to the heel region. This extensive damage likely leads to the generation of more glass particulates saturating the high pH solution. In this study, concentrated heat stress on the heel/base region of the vials was achieved by using a heat block with wells that allow the encapsulation of the base/heel region instead of an oven as used by Sloey et al. Through this modification, we generated glass particulates that were stable for several weeks in Vial A. This extended storage time potentially allows for a batch generation of such particulates that can serve as positive controls during release and stability testing, as well as for glass vial compatibility studies.

**Fig. 2:**
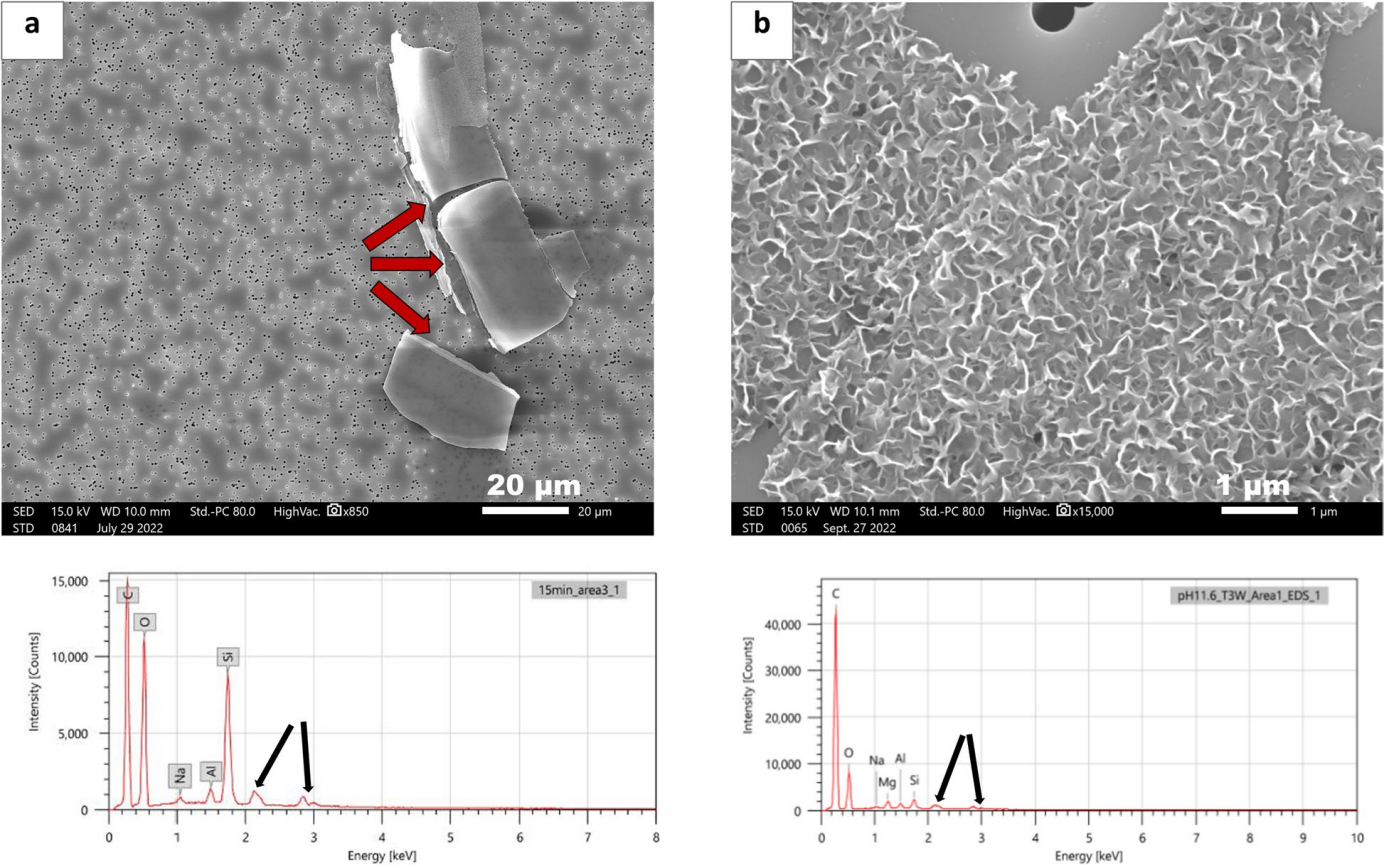
**a** SEM image in addition to EDX spectra of particulates extracted from Vial A (indicated with red arrows) heated for 15 min followed by exposure to pH 10, **b** SEM image in addition to EDX spectra of particulates extracted from Vial B treated with pH 11.6. The unlabeled peaks are from Au/Pd (indicated with black arrows) from sputter coating which we omitted during the analysis of the EDS spectra of each particulate

To generate particulates in Vial B, we followed the method developed by Ma et al. (Ma et al. 2021), employing 76 mM sodium glutarate solution at pH 11.6 (Ma et al. 2021). The particulates were generated within three weeks of incubation. Based on the particle morphology, the failure mode for particulates generated in Vial B is different from that obtained in Vial A, as shown in the panels of Fig.2. While Vial A undergoes delamination to generate flakes/lamellae (Fig. 2a), the particulates from Vial B captured on filters exhibit a “honeycomb-like” micro-surface roughness (Fig. 2b). The internal surface of Vial B shows deposition of particulates on the vial surface. In contrast, the inner surface of Vial A shows pitting and corrosion of the vial surface (Figure A1, Supplemental Information). The results presented in the panels of Figure A1 of the supplementary material are consistent with previous observations by Ma et al., who detailed the different mechanistic routes to the failure modes (Ma et al. 2021). The SEM-EDS analysis summarized in Table1 and spectra presented in the bottom panels of Fig. 2confirm that the particulates possess elemental compositions that are derived from their corresponding glass vial sources. Note that a detailed discussion of the mechanism of glass delamination has previously been described in the literature (Ma et al. 2021) and this paper focuses on the analytical microscope methods used to detect and evaluate glass particulates.

**Table 1:** Summary of elements obtained from EDS analysis of glass particulates from vial A and vial B

Element	Line	Mass %	Atom %
**EDS of Particulate from vial A**			
** C**	K	48.55 ± 0.07	57.95 ± 0.09
** O**	K	40.74 ± 0.13	36.51 ± 0.11
** Na**	K	4.48 ± 0.0.01	0.30 ± 0.01
** Al**	K	0.95 ± 0.02	0.51 ± 0.01
** Si**	K	9.28 ± 0.04	4.74 ± 0.02
**Total**		100.00	100.00
**EDS of Particulate from vial B**			
**C**	K	67.42 ± 0.06	74.26 ± 0.06
**O**	K	28.87 ± 0.11	23.87 ± 0.09
**Na**	K	0.26 ± 0.01	0.15 ± 0.01
**Mg**	K	1.20 ± 0.02	0.65 ± 0.01
**Al**	K	0.78 ± 0.01	0.38 ± 0.01
**Si**	K	1.47 ± 0.02	0.69 ± 0.01
**Total**		100.00	100.00

### Detection of thin glass particulates with optical microscopy

**Fig. 3:**
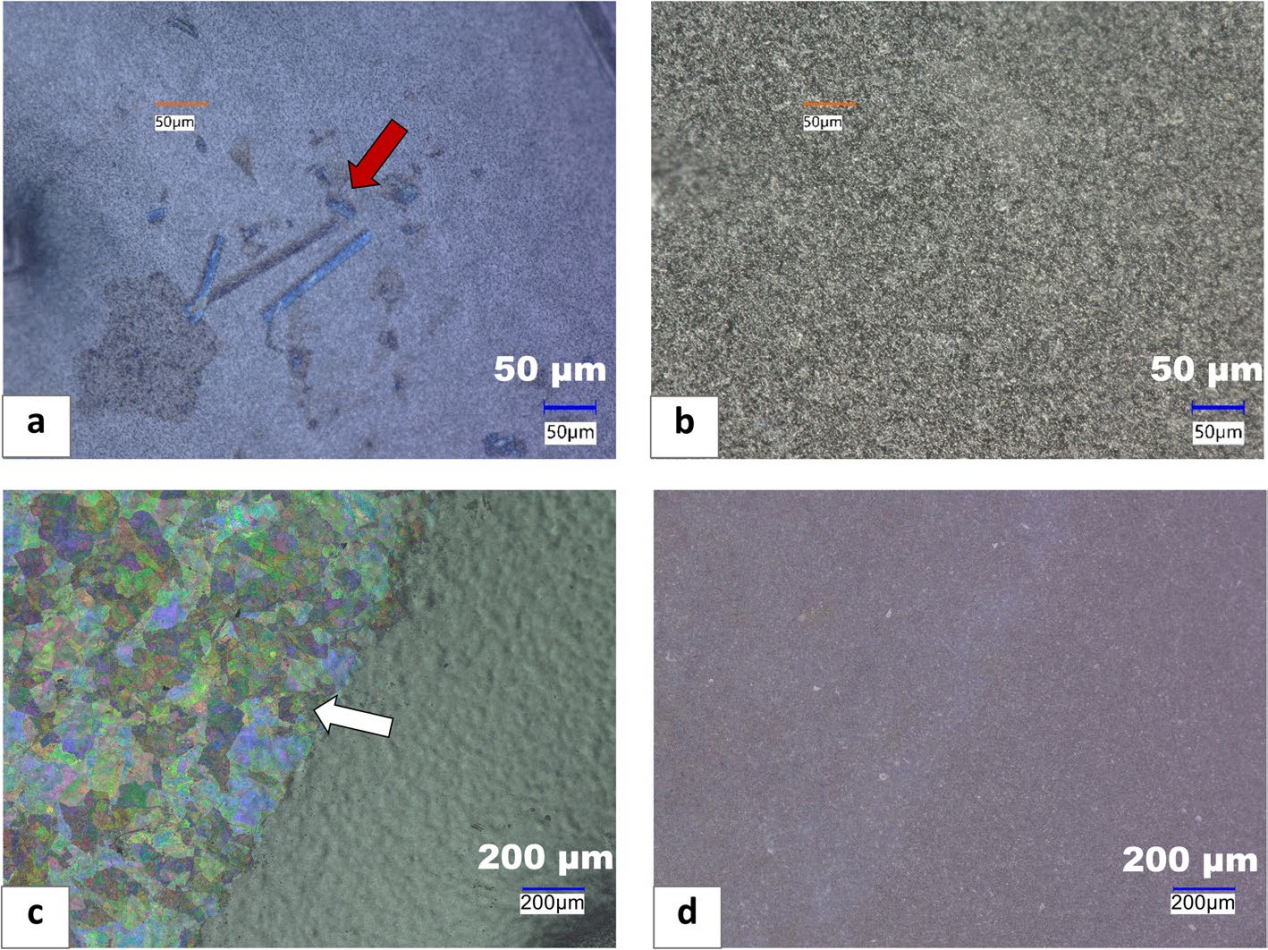
Keyence optical microscope image of: **a** glass particulates from Vial A under coaxial illumination exhibiting thin film interference (red arrow), **b** glass particulates from Vial A missing under oblique illuminations, **c** glass particulates from Vial B under coaxial illumination exhibiting thin film interference (white arrow), **d** glass particulates from Vial B missing under oblique illuminations

Employing OM with different illumination sources, such as coaxial and oblique, allows for the detection of thin particulates that are challenging to detect by other analytical techniques. Figure 3 shows the optical micrographs of particulates extracted from stressed vials using the methods detailed in the experimental section. The panels of Fig. 3a and b shows the micrograph of particulates from Vial A under both coaxial and oblique illuminations. A close inspection of Fig. 3a revealed that the particulates exhibit colors, that are absent in the micrograph in Fig. 3b. This coloration observed in the micrograph shown in Fig. 3a is from thin-film interference, described in more detail in the subsequent section. This interference occurs due to the thin nature of the particulates, which is on the order of the wavelength of visible light, allowing the constructive interference of light waves traversing through the particulates from multiple reflections (Urone 2012). Therefore, this analytical method provides early detection capabilities for thin glass particulates in parenteral products.

Additionally, the panels of Fig. 3c and d show the robustness of this microscopic technique as applicable to Vial B. Even though the mechanism of the failure modes in Vials A and B differ, we observed similar optical behaviors of the glass particulates generated in both vials under coaxial and oblique lighting. Furthermore, this approach enabled selective detection of thin glass particulates in the presence of other non-glass particles in the stressed vials. The thin glass particulates were observed with bright colors under coaxial lighting and were not visible under oblique lighting. The other non-glass particles (e.g., aluminum and rubber fragments) lacked the thin-film interference phenomenon observed in glass particulates under coaxial lighting. They were visible with high contrast under both oblique and coaxial lighting as shown in the panels in Figure A2a and b of the supplementary material. Therefore, applying this microscopic technique is valuable as an initial step in screening thin glass particulates prior to carrying out advanced analyses, such as electron microscopy. Hence, the application of OM, leveraging the principle of thin-film interference, is valuable in detecting thin glass particulates during the evaluation of container-product compatibility and the selection of product vials. In the subsequent section of this work, we describe this interference phenomenon and how it was applied in the detection of particulates obtained from the stressed Vial B.

#### Thin-film interference in the detection of particulates

Thin-film interference is a critical concept in many optics-related studies (Kitagawa 2020; Kitagawa 2013; Zhang et al. 2021; Fan et al. 2019; Sanni et al. 2019). However, its extensive application in detecting glass particulates during the pharmaceutical development of parenteral products, as exemplified in this work, remains largely limited. Interestingly, thin-film interference acts like a built-in contrast agent for an otherwise transparent material. The glass particulates under coaxial lighting may appear colored as if it was stained with dye(s), even though there was no sample preparation after filtration. The colors observed due to thin-film interference strongly depend on the light’s angle of incidence (AOI), refractive index, and thickness of the particulates (Urone 2012). In this section, we describe the angle-dependent microscopy experiments using coaxial and oblique illumination sources on both the stereomicroscope and digital microscope to demonstrate the application of thin-film interference in detecting glass particulates.

##### Detection of thin-film interference using the stereomicroscope

**Fig. 4:**
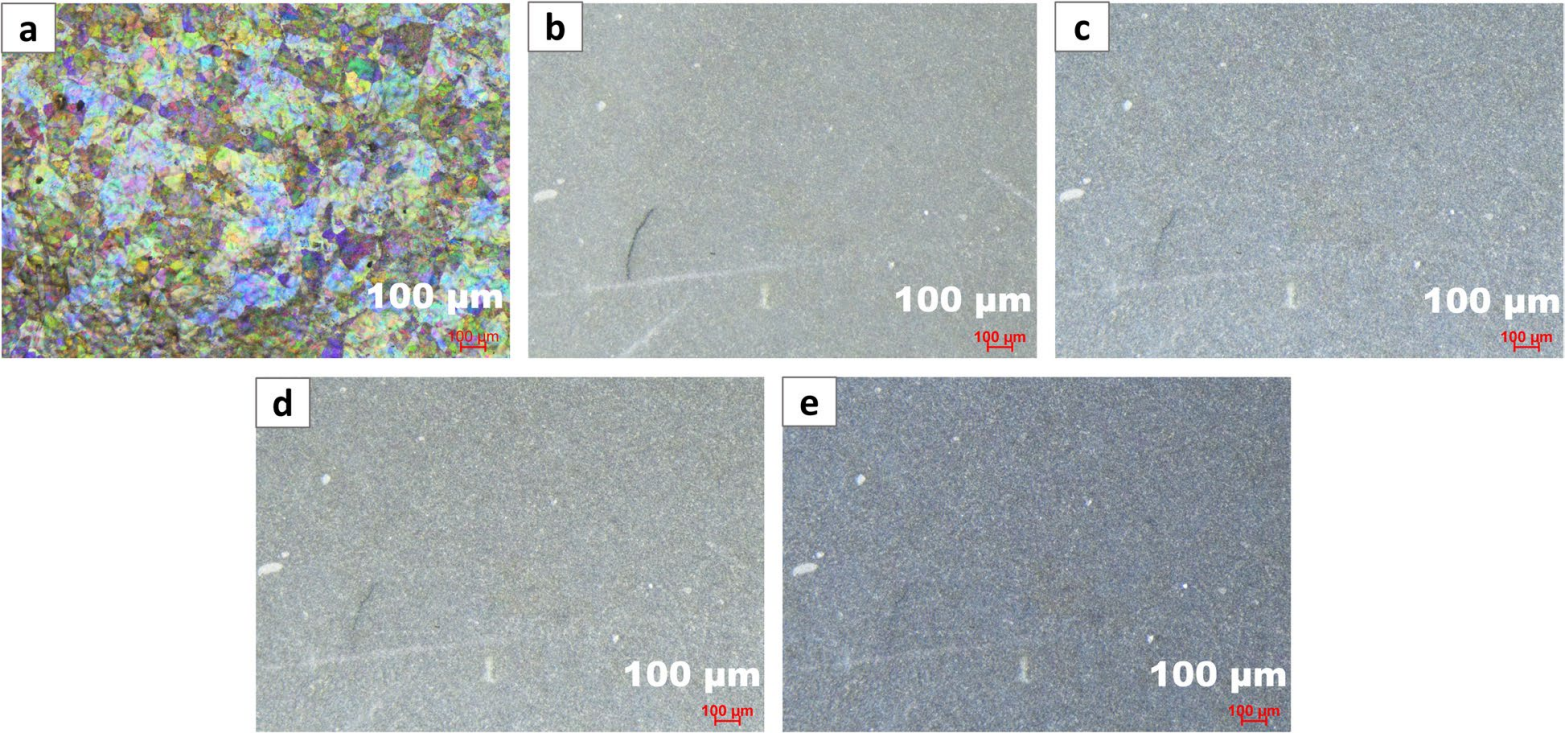
**a** Optical image of particulates under coaxial lighting showing thin-film interference, **b-e** Optical images of the same filter shown in panel a at 60, 45, 30, and 15º, oblique angles respectively showing the non-detection of thin film interference/ glass particulates. All images were acquired on the stereomicroscope

Within our instrumental setup, thin glass particulates were detected under coaxial illumination by observing thin-film interference, which disappeared at oblique angles in the range of 15–60º. The optical micrographs shown in Fig. 4a-e detail our findings from experiments centered on the angular dependence of thin-film interference using both coaxial and oblique illumination sources on a stereomicroscope. In this study, the AOI (defined relative to the sample stage) is represented in Fig. 1a. We varied the AOI on particulates from Vial B collected on polycarbonate membrane filters between 0 and 90º where the coaxial/episcopic illumination is 90º (through the objective) based on our definition in Fig. 1a. Our study revealed the detection of glass particulates under coaxial illumination, which disappears under oblique illumination. Detailed descriptions of oblique angle detection with information on optical paths, combination of diffracted and undiffracted rays to afford the detection of particulates have been previously discussed (McCrone 2024; Nikon 2024; Sanchez et al. 2018). Hence, this microscopic detection method using both coaxial and oblique lighting was successfully implemented using oblique lighting at angles 15–60º. The optimum performance angle range should be evaluated based on the available instruments and configuration in each laboratory setting.

##### Detection of thin-film interference using the digital microscope

**Fig. 5:**
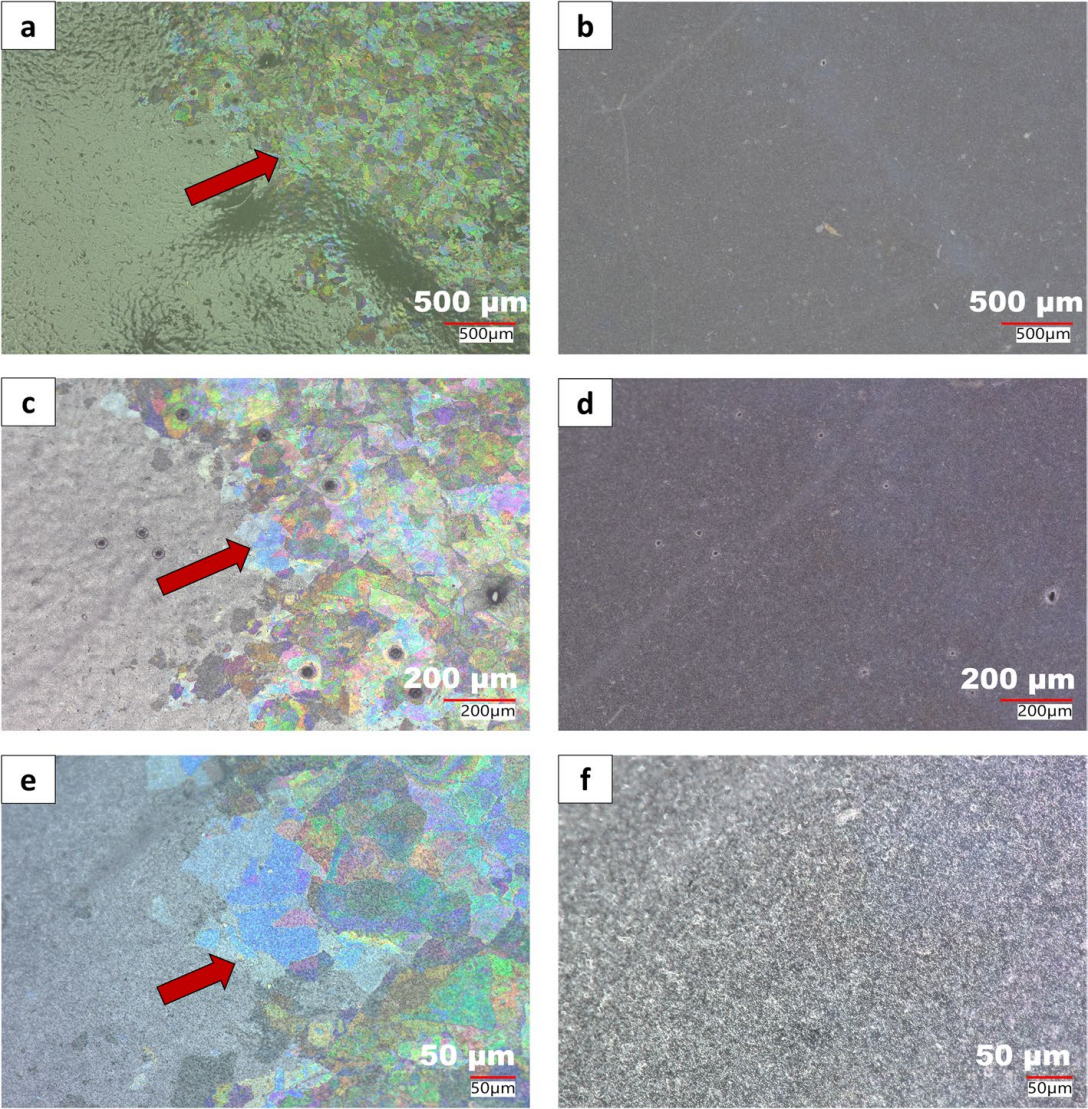
Keyence digital microscope optical images of particulates from Vial B exposed to pH 11.6 for 3 weeks at 60 °C, observed using **a** E20 objective lens at 80x magnification with coaxial illumination showing particulates exhibiting thin film interference (red arrow) **b** E20 objective lens at 80x magnification with oblique illumination (AOI: 48.1–59.5 °) showing absence of particulates exhibiting thin film intereference **c** E100 objective lens at 200x magnification with coaxial illumination showing particulates exhibiting thin film interference (red arrow) **d** E100 objective lens at 200x magnification with oblique illumination (AOI: 46.0-56.6 °) showing absence of particulates exhibiting thin film intereference **e** E500 objective lens at 500x magnification with coaxial illumination showing particulates exhibiting thin film interference (red arrow) **f** E500 objective lens at 500x magnification with oblique illumination (AOI: 18.5–22.0 °) showing absence of particulates exhibiting thin film intereference

Extending our experiment to include digital microscopes, as shown in the photomicrographs in panels of Fig. 5a-e, allows us to demonstrate the robustness of this analytical method in detecting thin glass particulates using OM with coaxial and oblique lighting. Notably, in the digital microscopes with oblique light sources built into the objective lenses (“ring lights” as shown in Fig. 1b), the oblique AOI cannot be freely chosen and is determined by the geometry of the objective lens. We measured and calculated the allowable incident angles on the Keyence to be between 18.5–59.5°, as detailed in Table A1 and Figure A3 of the supplementary material. The digital microscope’s range of oblique AOIs spanned a narrower range than explored with the gooseneck light source, and the observations under coaxial and oblique lighting were consistent with images collected on the stereomicroscope as evident in the images shown in Fig. 5a-f. This extension to other microscopes demonstrates the accessibility and robustness of this detection approach for identifying thin glass particulates in parenteral drugs resulting from failure modes due to chemical interaction with the inner wall of the glass vials. Therefore, microscopes available within a laboratory can be retrofitted for analysis without necessarily purchasing a new and state-of-the-art systems.

##### Other considerations when applying thin-film interference for particulate detection

**Fig. 6:**
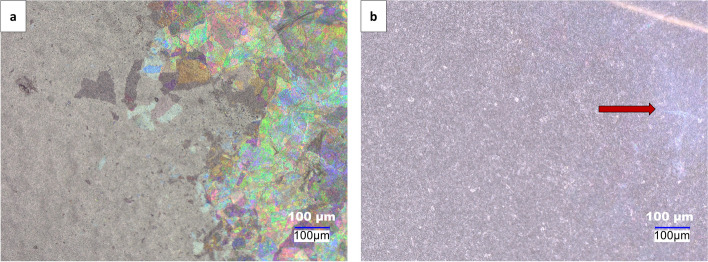
**a** Optical image of particulates extracted from Vial B treated with pH 11.6 showing the thin-film interference under coaxial illumination **b** Optical image of particulates extracted from Vial B treated with pH 11.6 showing the disappearance of thin-film interference, but low contrast features indicated using red arrow, under oblique illumination. Images were acquired on the Keyence digital microscope

An important consideration during the application of OM in the detection of glass particulates is the observation of thin-film interference under coaxial illumination, which is missing under oblique illumination. As previously highlighted throughout this work, using both coaxial and oblique illumination sources allowed us to determine that under normal sample mounting conditions within our experimental configuration, the oblique angles appropriate for the disappearance of thin-film interference from glass particulates on the filter membrane is between 15–60º. However, under certain circumstances, low contrast features of glass particulates in some portion of the filter may be observed under oblique illumination which are more difficult to visualize as compared to the image under coaxial illumination. An example of such a scenario is shown in Fig. 6a and b where the low contrast appearance in Fig. 6b is likely due to surface roughness or stacking of several glass particulates, which can occur during filtration. This roughness and stacking can cause the particulate to display different interference behaviors (Kats and Capasso 2014). The observed hue in Fig.6b is likely due to a phenomenon known as iridescence, which has been observed in other thin-film applications (Fan et al. 2019). Hence, combining the appearance of colored glass particulates as a result of thin-film intereference under coaxial illumination with the disappearance of the colored glass particulates under oblique illumination between angles 15–60º serves as an excellent screening test for thin glass particulates during glass vial compatibility studies. Despite the promising application of this microscopic approach in detecting thin glass particulates, it should only be employed as a complementary technique rather than a replacement for SEM-EDS, as valuable morphological and elemental information can be drawn from such studies. Additionally, as discussed subsequently, other spectroscopic techniques, such as infrared spectroscopy, can be coupled with this optical microscopy approach to gain insight into the identity of particulates.

### Complimentary detection and analysis of glass particulates

#### LIBS analysis

**Fig. 7:**
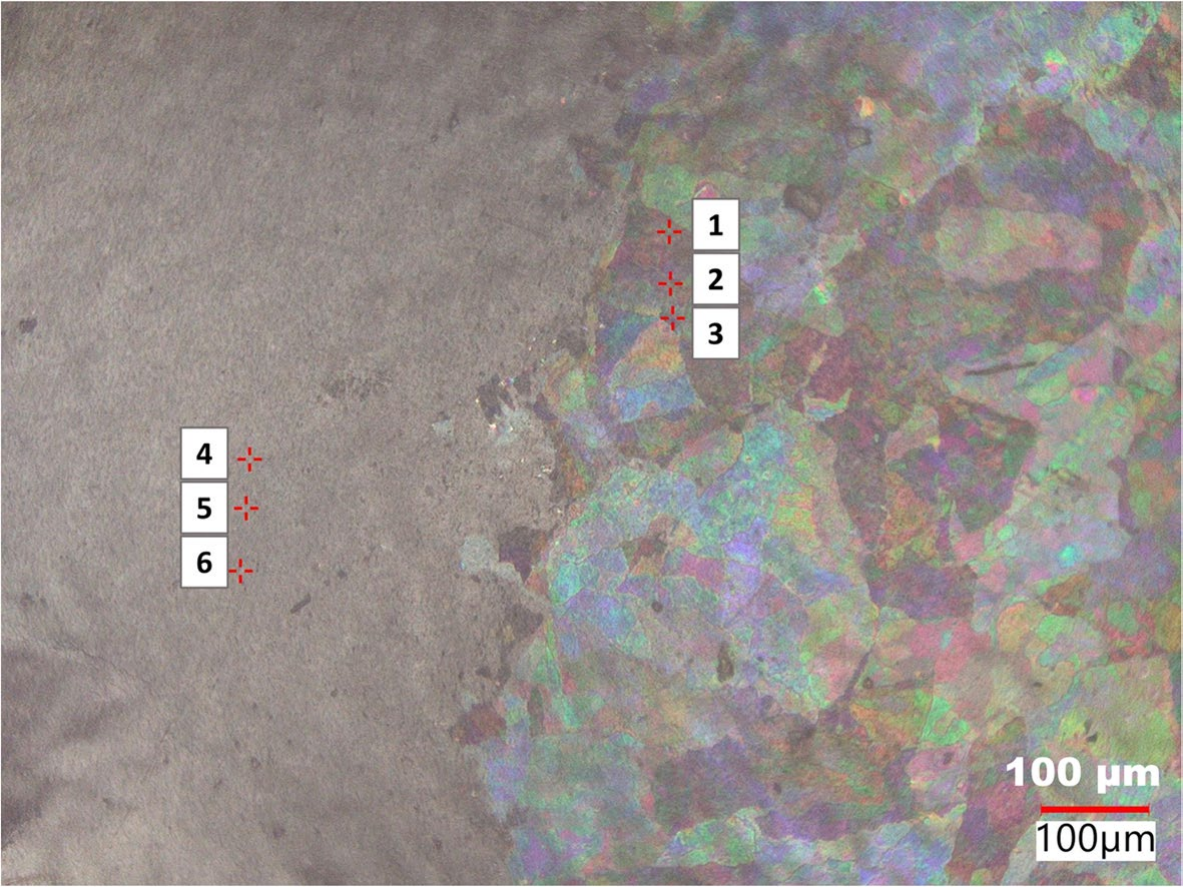
Optical micrograph showing the LIBS analysis of particulates extracted from Vial B treated with pH 11.6 showing the thin-film interference under coaxial illumination on the Keyence digital microscope. Spots 1,2, and 3 are due to the thin glass particulates, while spots 4, 5, and 6 are due to the membrane filter. The red crosshairs beside each spot number are the laser spots during analysis

The advent of state-of-the-art microscopes equipped with elemental analyzers with spot sizes down to a few microns potentially allows for rapid microscopy and elemental analysis to identify thin glass particulates such as those discussed in this paper. A critical aspect of this analysis is the ability to detect thin glass particulates under an optical microscope by the thin-film interference. LIBS has been used in various applications to identify various particles, including glass (Pontes et al. 2024; Gerhard and Hermann 2023). However, to the best of our knowledge, this paper demonstrates the first combination of thin-film interference and LIBS in detecting thin glass particulates generated due to the failure of pharmaceutical vials. The micrograph in Fig.7 shows the laser spots and glass particulates exhibiting thin film interference. The summary of the results from the LIBS analysis on particulates obtained from vial B is in Table 2. We observed the elements in the thin glass particulates (spots 1, 2 and 3) are different from those obtained from the polycarbonate membrane filter (spots 4, 5, and 6). We demonstrated the presence of elements such as Na, Mg, and Si only in the regions containing the thin glass particulates. In contrast, the background (membrane filter) shows no evidence of Na, Mg and Si content. The elements detected using LIBS are similar to those obtained from SEM-EDS as shown in Table 1. This analysis demonstrates how the LIBS analysis can be used in support of the evaluation of container compatibility in pharmaceutical vials.

**Table 2:** Summary of elemental composition (weight%) obtained from LIBS analysis of glass particulates from vial B

Spot	C (%)	O (%)	H (%)	Si (%)	Na (%)	Mg (%)	Total (%)
**1**	39.4	41.6	4.1	9.2	3.7	2.0	100
**2**	52.1	32.3	4.4	5.4	2.8	3.0	100
**3**	42.0	39.8	4.5	6.7	4.3	2.7	100
**4**	66.7	28.3	5.0	ND	ND	ND	100
**5**	68.3	26.8	4.9	ND	ND	ND	100
**6**	72.1	23.4	4.5	ND	ND	ND	100

*ND* Not detected

#### FTIR microspectroscopy analysis

Coupling a spectroscopic technique, such as FTIR, to an optical microscope allows for rapidly detecting and characterizing glass particulates. This approach allowed us to determine that the particulate studied in this work is a silicate material.

**Fig. 8:**
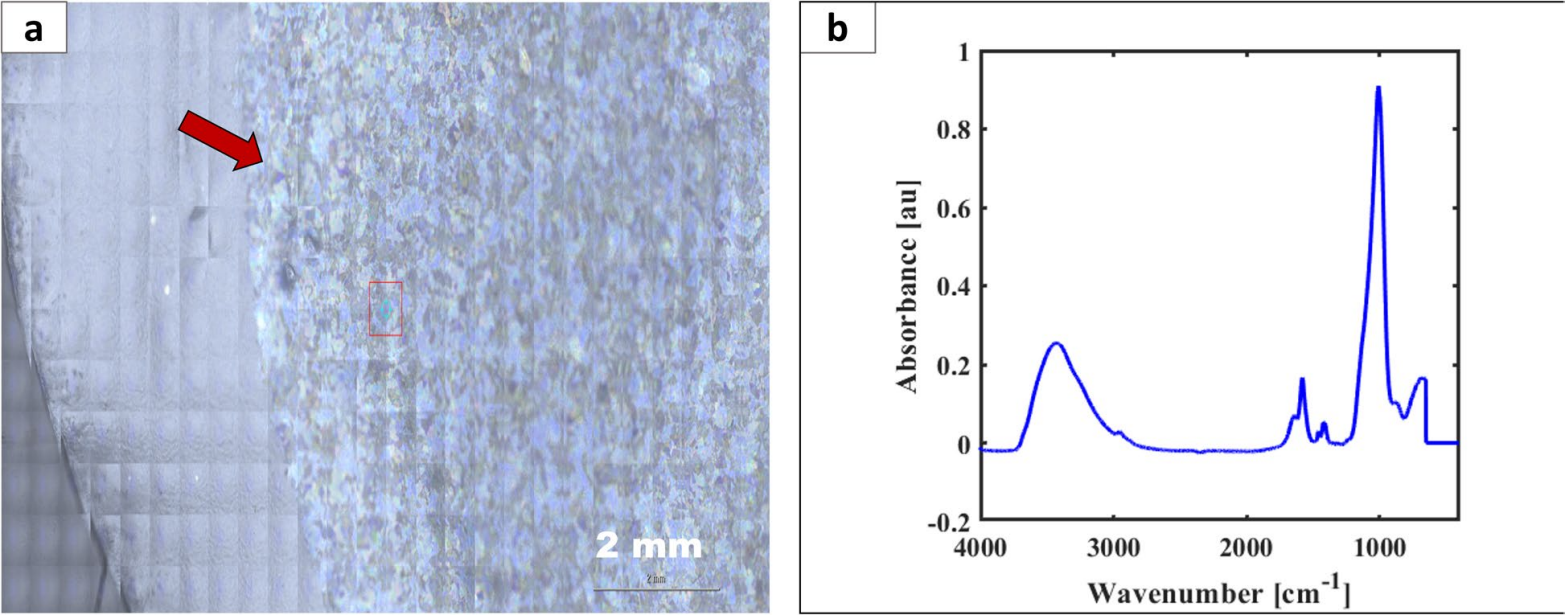
**a** Mosaic image of a portion of filter acquired on an FTIR RaptIR microscope using 15x objective, showing the particulates obtained from glass vial exposed to pH 11.6 (Vial B) possess thin film interference as indicated with the red arrow. **b** Infrared spectrum of particulate obtained from Vial B exposed to pH 11.6 obtained using the FTIR continuum microscope operating in transmission mode. Identical FTIR spectrum using the reflection and ATR modules of the RaptIR is shown in Figure A4b abd A4c of the supporting material

We used an FTIR microscope to acquire mosaic images of a portion of the membrane filter containing the particulates under reflection illumination (similar to coaxial lighting) on the Thermo RaptIR microscope. The mosaic image of the filter is shown in Fig. 8a, indicating a portion of the filter containing the glass particulate, acquired using the 15x objective of the FTIR microscope. The mosaic of the full filter acquired with the 4x objective is shown in Figure A4a of the supplementary material. Upon inspection of the images in Fig. 8a and A4a, we found that the optical characteristics (thin-film interference) of the particles observed under the Keyence digital microscope and stereomicroscope were preserved. Hence, we acquired the IR spectrum of the particulates first using the transmission mode on an FTIR continuum microscope, as detailed in the methods. The result of this analysis is shown in Fig. 8b. Second, using the reflection mode of the FTIR microscope with particulates on a gold membrane filter, we acquired the spectrum of particulates as shown in Figure A4b of Supplementary Material. Finally, the ATR module was used on the RaptIR microscope and the spectrum shown in Figure A4c of the Supplementary Material was obtained. This experiment demonstrates the detection of thin glass particulates by FTIR microscope. Importantly, it demonstrates the flexibility to perform FTIR microspectroscopy analysis in multiple ways. We note that for identification in a circumstance where the glass particulate is easily isolated, analysis by transmission mode is very useful. However, when particulates cannot be readily isolated from the membrane filter, direct analysis of particulates on a gold coated membrane filter by reflection or using the micro-ATR mode for analysis is very valuable. Following the analysis, we find that the spectral features recovered from the analysis of our thin glass particulate are similar to that of a silicate material earlier reported (Ratnaswamy et al. 2014)and other natural silicates (Kloprogge and Ponce 2021), which may precipitate in solution. This precipitation stems from the initial dissolution of elements in solution reaching a saturation point (Ma et al. 2021). In particular, the intense peak near 1000 cm^−1^ is due to the Si-O stretch, while those near 1650 and 3428 cm^−1^ are due to OH bending and stretching vibrations.

Our work highlights the rapid detection and characterization of these thin particulates using FTIR micro-spectroscopy. Hence, microspectroscopic analysis of the thin glass particulates described in this work enabled rapid detection and characterization. Therefore, implementing the characterization techniques detailed in this work allowed us to develop a rapid and specific approach for detecting and identifying thin glass particulates in parenteral samples. This complimentary microspectroscopic detection of particulates may aid the creation of internal libraries during vial assessment studies in parenteral products to support release, stability, and routine testing.

## Conclusions

This paper demonstrates the application of optical microscopy for detecting glass particulates generated in glass vials in contact with the parenteral drug products. The procedure highlighted for generating stable glass particulates as positive controls can serve as a reference to be incorporated in analytical methods to enable product development and commercialization. Using the analytical methodology in this paper, very thin glass particulates that would have been otherwise difficult to detect were distinguished from intrinsic or inherent non-glass particulates by employing coaxial and oblique lighting on an optical microscope. Additionally, we conclude that within our microscope configurations, thin-film interference is fully observable under coaxial illumination and disappears under oblique illuminations at angles between 15–60º. Finally, SEM-EDS, LIBS and FTIR microspectroscopy allowed us to characterize the precipitated particulates and determine that they are silicate materials originating from the parent glass vial. Hence, we developed an analytical microscopy method complementary to SEM, which can be used to detect and identify glass particulates in parenteral drug products to support packaging selection and quality control strategies.

## Supplementary Information

Supplementary Material 1.

## Data Availability

Data associated with this publication are available upon reasonable request.

## Abbreviations

OM: Optical microscopy
ATR: Attenuated total reflectance
AOI: Angle of incidence
FTIR: Fourier transform infrared
LIBS: Laser-induced breakdown spectroscopy
SEM-EDS: Scanning electron microscopy-Energy dispersive spectroscopy
