# Supplementary material for: Analytical microscopy techniques using coaxial and oblique illuminations to detect thin glass particulates generated from glass vials for parenteral drug products
Source: Appl Microsc. 2024 Oct 23;54:9. doi: 10.1186/s42649-024-00101-3 (PMC11499564; doi:10.1186/s42649-024-00101-3)
Supplement: Supplementary file 1 — Supplementary Material 1. [file 42649_2024_101_MOESM1_ESM.docx]

Supporting Information for

**Analytical Microscopy Techniques Using Coaxial and Oblique Illuminations to Detect Thin Glass Particulates Generated from Glass Vials for Parenteral Drug Products**

Adedayo M. Sanni^a^, Adedamola A. Opalade^a^, Armen Shamirian^a¥^, Spencer Mattson^b^, Eric Driscoll^b¶^, Michael St. Martin^b^, Shikhar Mohan^a^, Brooke Trimmer^a^, Tarq Bunch^b^, Robert Ovadia^c^, Jungjoo Yoon^a^, Sarina Ma^d^, Chris Foti^a^*.

^a^ Analytical Development and Operations, Gilead Sciences Inc., Foster City, CA 94404, USA

^b^ Development and Technical Services, Gilead Sciences Inc., La Verne, CA 91750, USA

^c^ Device Development and Clinical Packaging Engineering, Gilead Sciences Inc., Foster City, CA 94404, USA

^d^ Pharmaceutical Development and Technology, Gilead Sciences Inc., Foster City, CA 94404, USA

^¥^ Now at ORIC pharmaceuticals.

^¶^ No longer at Gilead Sciences.

| **Index** | **Page** |
| --- | --- |
| Figure A1: a) SEM image surface of vial A showing pitting and b) SEM image of inner surface of vial B showing microsurface roughness. | A2 |
| Figure A2: a) Optical image of non-glass particle (red arrow) observed under coaxial illumination, lacking the thin film interference b) Optical image of non-glass particle (red arrow) observed under oblique illumination with high contrast. Indicating that these particles respond differently from the glass particulates described in the main text of the manuscript. | A3 |
| Figure A3: Measurement of working distance, ring inner/outer diameter, and **c**alculation of the AOI on the Keyence microscope as detailed in Table A1 | A4 |
| Table A1. Calculated Keyence digital microscope ring (oblique) lighting geometry | A5 |
| Figure A4: **a)** Mosaic images of the full membrane filter containing the filtered glass particulates (indicated with red arrow) from vial B acquired on an FTIR RaptIR microscope using the 4x objective with reflected light. **b)** FTIR spectrum of thin glass particulates from vial B on a gold membrane filter acquired using the reflection mode of the RaptIR microscope showing the spectra signatures are similar to that reported in Figure 8b of the main article. **c)** FTIR spectrum of thin glass particulates from vial B acquired with the attenuated total reflectance module of the RaptIR microscope showing the spectra signatures are similar to that reported in Figure 8b of the main article. The negative peaks are from subtraction of the membrane filter spectrum from the sample + membrane spectrum. This analysis showed that RaptIR FTIR microscope can be used to acquire spectra rapidly without any sample preparation. | A6 |


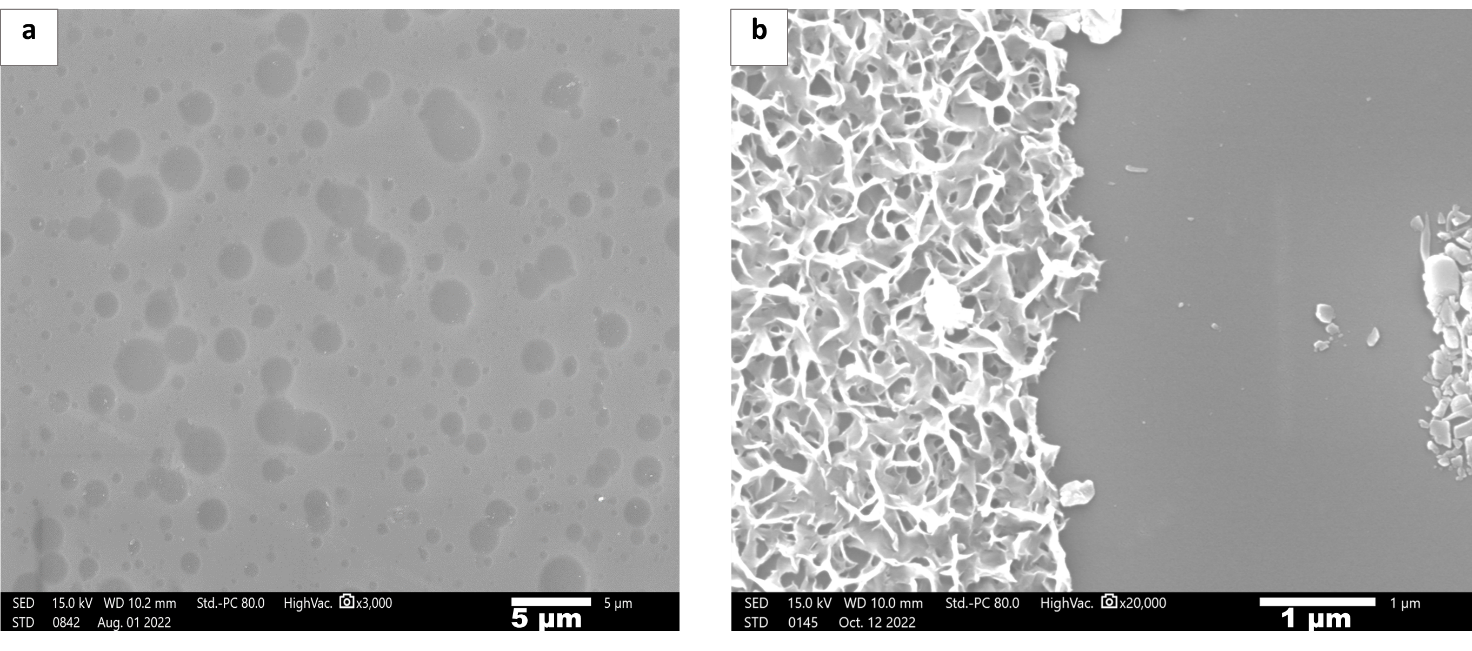


**Figure A1**: SEM image surface of vial A (left) showing pitting and SEM image of inner surface of vial B (right) showing microsurface roughness.


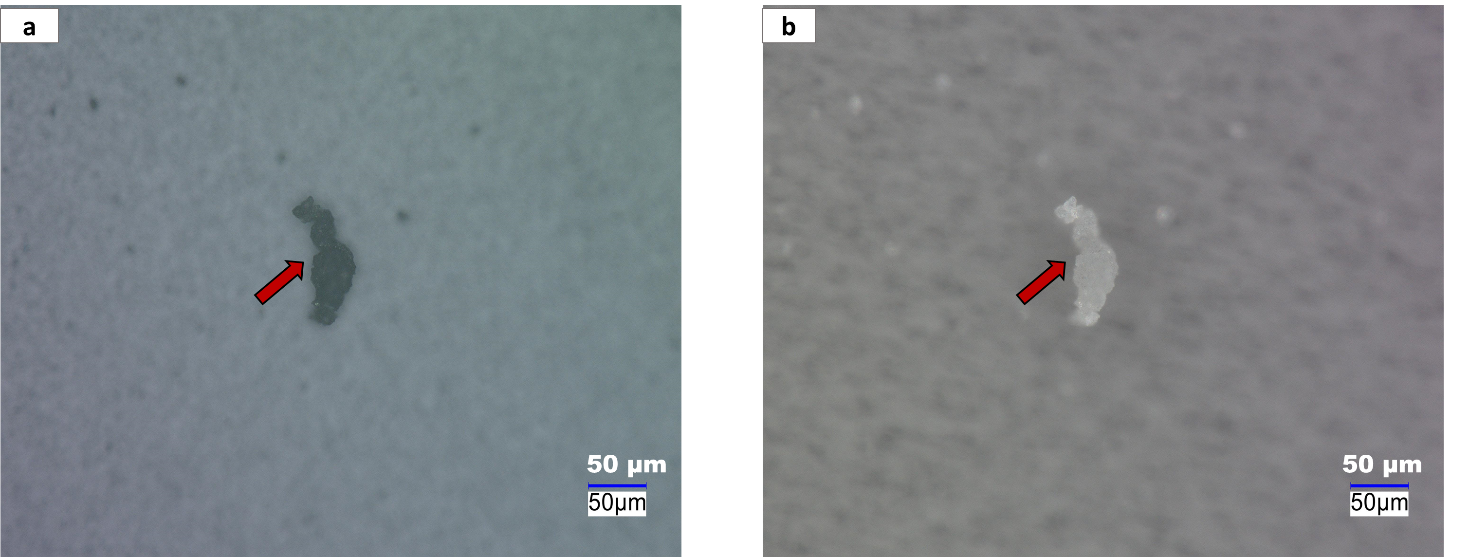


**Figure A2**: **a)** Optical image of non-glass particle (red arrow) observed under coaxial illumination, lacking the thin film interference **b)** Optical image of non-glass particle (red arrow) observed under oblique illumination with high contrast. Indicating that these particles respond differently from the glass particulates described in the main text of the manuscript.

On the Keyence digital microscope, the ring light (oblique) AOI is fixed for each objective lens and is a function of working distance (W) and the ring inner diameter (I.D.) and outer diameters (O.D.). The region being imaged will be illuminated with rays between these min and max AOIs. The dimensions shown in Table A1 were measured for each objective lens using digital calipers and are summarized below.


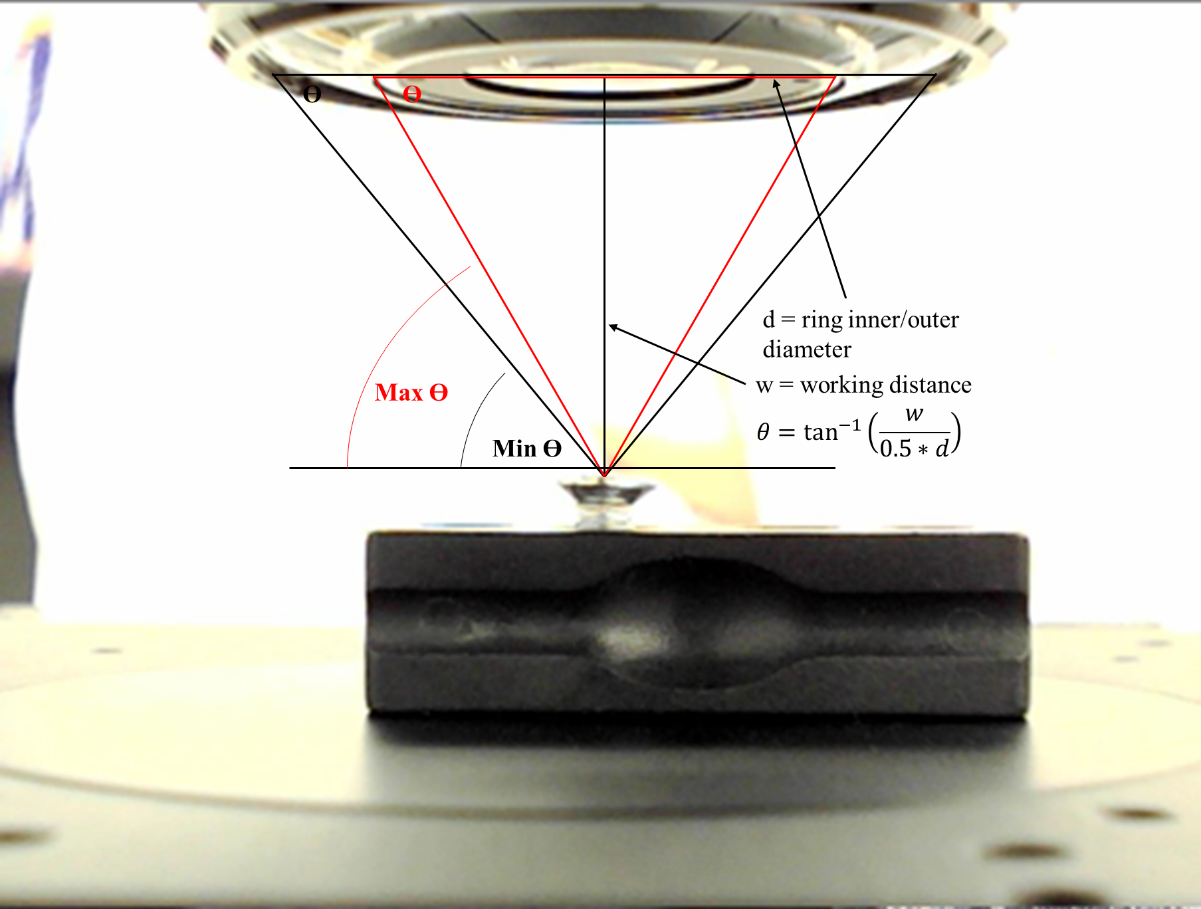


**Figure A3:** Measurement of working distance, ring inner/outer diameter, and **c**alculation of the AOI on the Keyence microscope as detailed in Table 1.

**Table A1. Calculated Keyence Digital Microscope Ring (Oblique) Lighting Geometry**

| **Objective Lens** | **Magnification** **Range** | **W (mm)** | **Ring I.D. (mm)** | **Ring O.D. (mm)** | **Min AOI (degrees)** | **Max AOI (degrees)** |
| --- | --- | --- | --- | --- | --- | --- |
| E20 | 20X-100X | 31.0 | 36.5 | 55.6 | 48.1 | 59.5 |
| E100 | 100X-500X | 21.2 | 28.0 | 41.0 | 46.0 | 56.6 |
| E500 | 500X-2500X | 5.9 | 29.2 | 35.3 | 18.5 | 22.0 |

Optical microscopy images of the particulates obtained from vial B exposed to pH 11.6 taken with the Keyence digital microscope are provided at three different magnifications and AOIs shown in Table A1. At each magnification, the sample was imaged under coaxial illumination and ring/oblique illumination, as shown in Figure 5 of the main article.

.

**
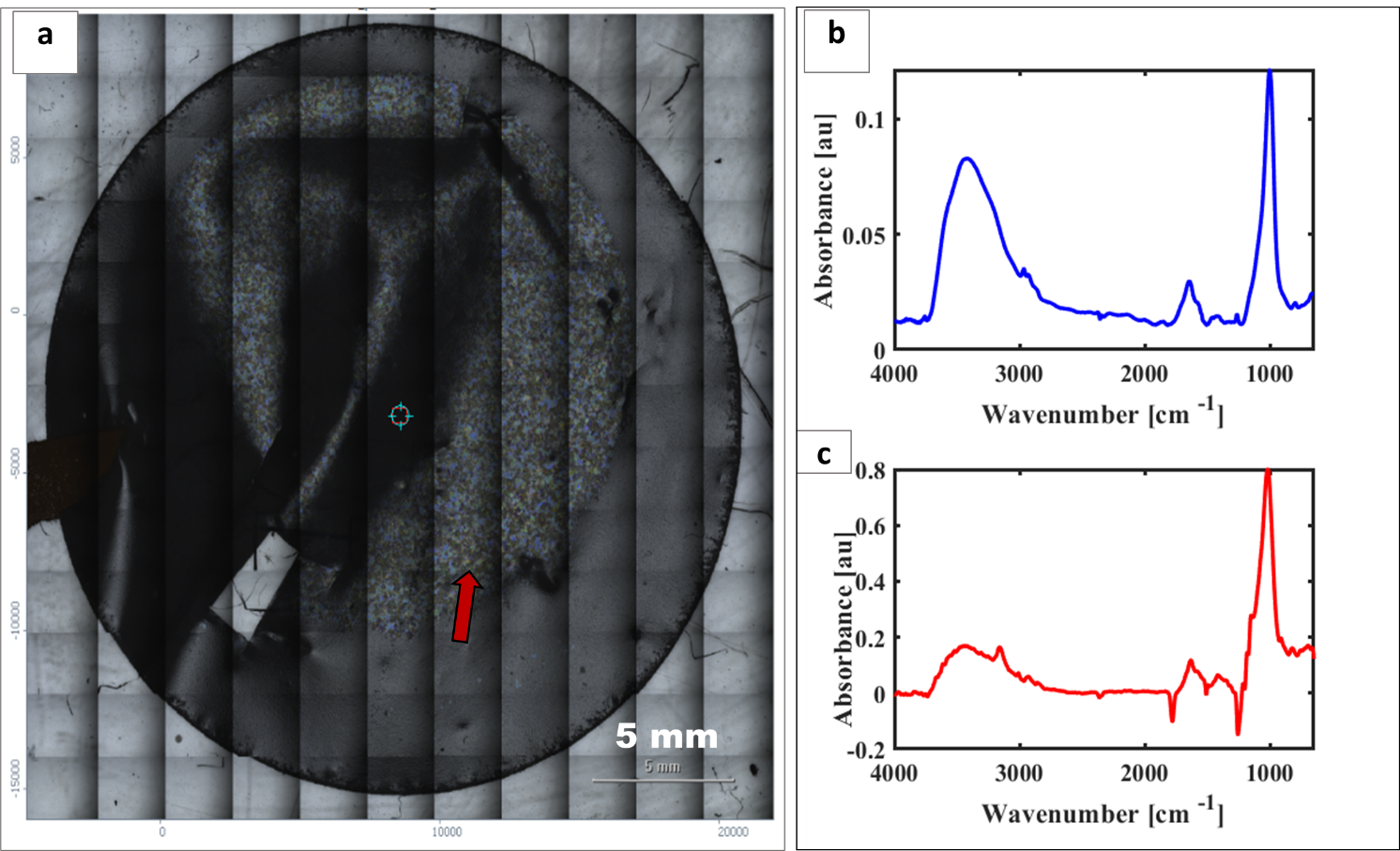
**

**Figure A4**: **a)** Mosaic images of the full membrane filter containing the filtered glass particulates (indicated with red arrow) from vial B acquired on an FTIR RaptIR microscope using the 4x objective with reflected light. **b)** FTIR spectrum of thin glass particulates from vial B on a gold membrane filter acquired using the reflection mode of the RaptIR microscope showing the spectra signatures are similar to that reported in Figure 8b of the main article. **c)** FTIR spectrum of thin glass particulates from vial B acquired with the attenuated total reflectance module of the RaptIR microscope showing the spectra signatures are similar to that reported in Figure 8b of the main article. The negative peaks are from subtraction of the membrane filter spectrum from the sample + membrane spectrum. This analysis showed that RaptIR FTIR microscope can be used to acquire spectra rapidly without any sample preparation.
